# The hexagonal shape of the honeycomb cells depends on the construction behavior of bees

**DOI:** 10.1038/srep28341

**Published:** 2016-06-20

**Authors:** Francesco Nazzi

**Affiliations:** 1 Dipartimento di Scienze AgroAlimentari, Ambientali e Animali, Università degli Studi di Udine, via delle Scienze 206, 33100 Udine, Italy

## Abstract

The hexagonal shape of the honey bee cells has attracted the attention of humans for centuries. It is now accepted that bees build cylindrical cells that later transform into hexagonal prisms through a process that it is still debated. The early explanations involving the geometers’ skills of bees have been abandoned in favor of new hypotheses involving the action of physical forces, but recent data suggest that mechanical shaping by bees plays a role. However, the observed geometry can arise only if isodiametric cells are previously arranged in a way that each one is surrounded by six other similar cells; here I suggest that this is a consequence of the building program adopted by bees and propose a possible behavioral rule ultimately accounting for the hexagonal shape of bee cells.

The hexagonal shape of the cells of the bees’ honeycomb has intrigued laypeople and scientists for ages. Why do bees build this kind of cells? How can they achieve such a spectacular structure? Early natural philosophers, like Marcus Terentius Varro^1^, based on the observation that hexagons possess the highest surface/perimeter ratio, compared to other polygons that can be used for tiling the plane, suggested that honey bees build their hexagonal cells in order to achieve the best economy of material. Eighteen centuries later, Darwin surpassed the early teleological explanations noting that the swarm wasting least honey in the secretion of wax would have succeeded best^2^. Precise data about food conversion rates in the production of beeswax^3^ support the intuition of the great naturalist.

However, for a complete understanding of a given phenomenon, both the proximate and ultimate sets of causes have to be explained and interpreted^4^; in other words, both the why and the how questions need to be addressed.

Varro suggested the correspondence between the bees’ legs number and the angles of the comb cell might hint of a possible construction method^1^; in any case, the geometers’ skills of bees were not questioned for a long time. However, cells are not hexagonal when they are built but rather circular, as already suspected by ancient observers and easily demonstrated by studying wax combs in the early stages of construction^5^,^6^. Rasmus Bartholin suggested that hexagons would result automatically from the pressure of each bee trying to enlarge as much as possible each cell^7^, while Thompson argued that it is surface tension that molds the soft wax into a hexagonal pattern of cells^8^, as a result of the formation of three 120° angles between the wax walls converging in a triple junction. More recently, Pirk and collaborators provided support to this latter hypothesis by studying the wax condensing around an array of rubber bungs^5^, while Karihaloo and coworkers reached a similar conclusion by modelling the effect of surface tension at the triple junction between adjacent cells^6^. This latter evidence, supporting the interpretation of the honeycomb architecture as the result of blind physical forces rather than biological engineering, lead Ball to state that there does not seem to be much room left for the honey bees’ engineering prowess^9^. However, a recent study questioned the possibility that surface tension alone can explain the process. In particular, Bauer and Bienefeld^10^ argued that bees do not heat up wax to the temperatures needed for the wax to reach the liquid equilibrium and proposed an alternative explanation based on the mechanical shaping of cells. In any case, available data about the thermal properties of beeswaxes^11^ indicate that the onset of the wax melting temperature is very close to the range observed by Bauer and Bienefeld^10^. In conclusion, the formation of the hexagons is still a matter of debate and a definitive conclusion has not yet been reached.

Anyway, regardless of the mechanism leading to the hexagonal shape of the primitively circular cells, an array of hexagonal prisms can be obtained with any of such mechanisms only if isodiametric cylinders are previously arranged in a regular tightly packed array, with each cylinder surrounded by six other same sized cylinders.

In sum, despite the considerable attention it recently attracted, the mechanism through which hexagons are formed appears to be of secondary importance; instead, the central point appears to be the formation of the hexagonal pattern of isodiametric cylinders. Under this perspective, the questions to be addressed are:

1. Is a hexagonal pattern of cylinders an essential prerequisite for the formation of hexagonal cells?
2. How do bees build cylinders of exactly the same size?
3. How do bees arrange the cylinders in a hexagonal pattern?

Several methods have been exploited so far for the study of the building behavior of bees, and Darwin himself dedicated to the subject some interesting pages of the “Origin of the species”^2^. However, many bees are involved in this behavior normally occurring, under natural conditions, within a dark cavity. This makes the observation somehow more difficult than in the case of other hymenopterans that build nests made of hexagonal cells as well (see, for example, the studies carried out by West-Eberhard on paper wasps^12^). For this reason, some clever expedients were adopted, ranging from the use of painted wax mentioned by Darwin^2^ to the thermographic observation of building bees while repairing their nest altered by the careful intervention of the experimenter^10^.

In this study I try to address at best the above questions by using only methods that allow the study of such a complex behavior while minimizing the possible perturbation of the process under observation.

## Results and Discussion

**Figure 1:**
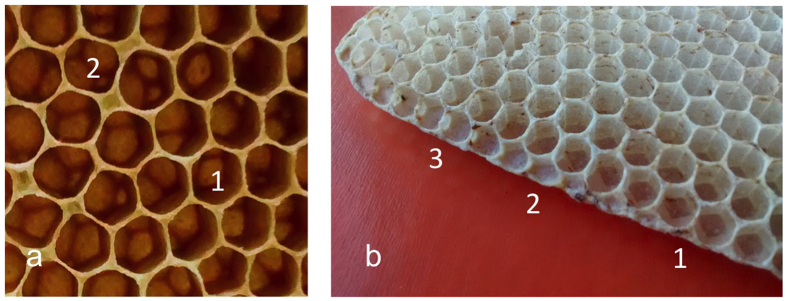
Geometry and construction of the honeycomb. (**a**) The pattern of cells influences their final shape: cell 1, surrounded by six other cells, has a hexagonal shape, with 120° angles between sides, whereas cell 2 has a different shape, with 90° angles between some consecutive sides. (**b**) The margin of a comb with stubs of cells at different stages of construction suggests a possible construction scheme (1: the construction of the cell base is started in the groove between two pre-existing cells; 2: when the cell base is as large as the cell diameter, the walls are started; 3: the walls encircle the first stub of the cell).

To see if the arrangement of cylindrical cells in a hexagonal pattern, with each cell surrounded by six other cells, is an essential prerequisite for the formation of the hexagonal cells, I carried out a systematic observation of honeycombs, searching for possible imperfections that could reveal the consequences of building errors involving the reciprocal arrangement of cells. This allowed to record a limited number of building errors; in these few cases, it was noted that when cells are not surrounded by six other cells, their final shape is not hexagonal but rather matches that of a polygon with as many sides as the number of surrounding cells (Fig. 1a and Fig. S1 for more examples). This indirectly confirms the influence of the pre-existing pattern on the final shape of cells.

Since previous work showed that bees use the body parts for measuring distances^3^, to test whether the capacity to build cylinders of the same size is within the possibility of bees, I compared the coefficient of variation of the honeycomb cells with that of the bees’ body parts. The coefficient of variation of the cells of a honeycomb, as measured in this study, was 2.1 ± 0.6% (Supplementary data file 1), which appears remarkably similar to the coefficient of variation reported by Ruttner for the hind leg and the proboscis of *Apis mellifera carnica*, that are, for example, 2.4 and 2.0% respectively^13^. Although the body parts that are used by bees to measure distances have not yet been definitely identified, there is abundant observational evidence regarding the possible role of antennae. Therefore, I also calculated the coefficient of variation of the flagellum of the antennae of bees collected from the same apiary where the field observations were carried out. The coefficient of variation was 1.5 ± 0.7%, which appears to be compatible with that of comb cells (Supplementary data file 2). In conclusion, at least in principle, the hypothetical bees’ “ruler” seems to be adequate for the purpose. On the other hand, the capacity of bees to “measure” distances is demonstrated by several evidences such as for example, the deposition of either haploid or diploid eggs by the laying queen into comb cells, according to their size^14^, or the regular spacing of adjacent combs in the nest, obtained by the building workers^3^.

Before addressing the last question, regarding the method used by bees to arrange the cylinders in a hexagonal pattern, it must be noted that bees don’t actually assemble preexisting objects but rather form a complex structure by progressively adding small wax scales produced and manipulated by many comb builder bees^3^. Given the difficulties of observing such process with minimally invasive methods, that do not alter the behavior to be observed, I resorted to the observation of wax combs under construction, as they appear after removing bees at different times.

The cross section of the wax comb under construction, with its thickness gradually declining from the center to the edges, reveals that cell construction starts from the pavement of cells, which are progressively extended in depth by adding more material around the cell rim. A careful inspection of the comb margin, with the stubs of cells at different stages of construction (Fig. 1b and Fig. S2 for more examples), suggests that the construction of the cell base may start in the groove between two adjacent, preexisting cells. When the base reaches a given size (i.e. when it is as large as the cell diameter), the lateral walls are built, eventually producing the first stub of the cell (Fig. 1b and Fig. S2). The addition of a new cell between two pre-existing ones generates two triple junctions that eventually acquire the regular appearance expected through either the liquid equilibrium process^5^ or any possible alternative mechanism^10^.

Overall, the rule suggested by this preliminary observation involves the gradual enlargement of the cell bottom, starting from the groove between two pre-existing cells and the construction of the cell walls as soon as the cell base reaches a certain size (Fig. S3).

In any case, comb construction is the result of a collective behavior, involving hundreds of individuals, whereby no central control structure exists and individuals follow simple rules related to the structuration of the environment, in a way that the environment influences the behavior, which in turn changes the environment; a mechanism that is known as “stigmergy”, according to the definition coined by Grassé^15^. Overall, the process involves a linear series of if-then decisions that can be well modeled using computer programs^16^. In fact, using similar methods, the architecture of the nest of several wasp species was studied, obtaining “in silico” constructions that appeared to be very similar to those observed in nature^17^.

**Figure 2:**
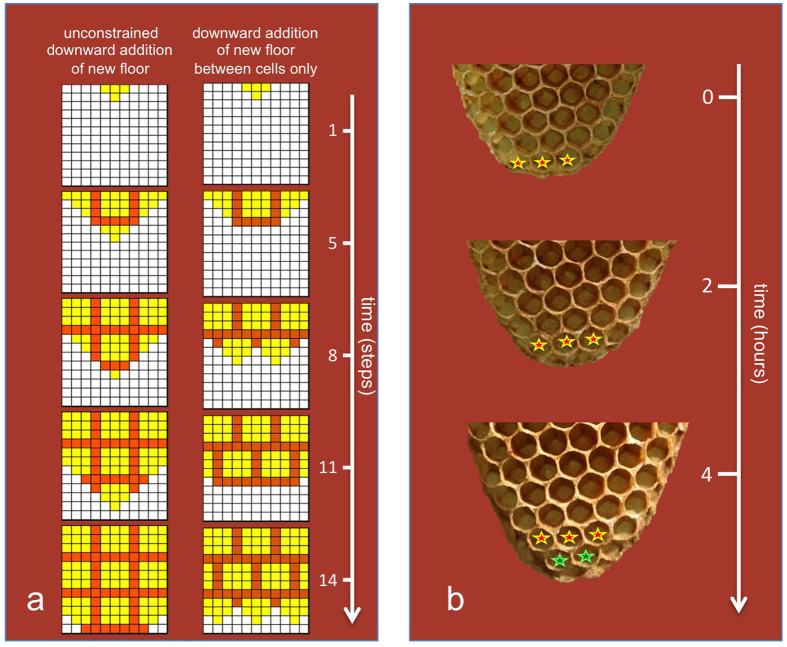
Growth of virtual and natural combs. (**a**) Results of a computer simulation performed using the spreadsheet provided (Supplementary excel file 1): the left and right columns of schemes represent the results obtained using two alternative sets of behavioral rules. In both cases, cell wall (orange squares) was added as soon as the adjacent floor (yellow squares) reached a certain size in either direction; on the left, the downward growth of the floor was permitted in contact with either floor or wall; on the right, to simulate the behavioral rule explained in the text, the downward growth of the floor was permitted only in contact with floor or a stretch of wall separating two adjacent cells. (**b**) Three images of the terminal portion of a comb, taken at 2 hours intervals, showing the cells added at each step.

Indeed a simple computer simulation incorporating the rules described above to build a simplified virtual comb made of quadrangular cells (Fig. S4) lead to an alternated pattern of similar sized cells (Fig. 2a; Supplementary excel file 1); ultimately, the alternated pattern accounts for the hexagonal shape of the cells because of any of the already mentioned mechanisms.

Conversely, if another behavioral rule is used, whereby the addition of cell floor can start anywhere, without being confined in the groove between adjacent cells (Fig. S4), an array of aligned cells is obtained (Fig. 2a; Supplementary excel file 1).

In order to see if the proposed building rule is actually exploited in the construction of combs by bees, sequential pictures of honeycombs under construction were taken at two hours intervals. The study of the tip of such combs revealed a growth pattern similar to that postulated here (Fig. 2b and Fig. S5 for further examples).

Honeycombs are made of two layers of contiguous cells, each one sharing its closed end with three cells of the opposite side (Fig. S6a); the perfect matching of the two sides of the comb is another feature that attracted remarkable attention for its geometrical implications and it is still waiting for an explanation. Despite the study of this further feature was outside the scope of this work, it is worth noting that the rule described above seems to be consistent with the observed geometry, provided that the two sides of the honeycomb grow in synchrony and the beginning of the construction of the cell base, in the groove between two adjacent cells on one side of the comb, coincides with the construction of the lateral walls of a cell on the opposite side (Fig. S6b).

In conclusion, regardless of the mechanism transforming the close packed cylinders into hexagonal prisms, here I draw the attention to the causes responsible for the geometrical pattern of cylinders on which that mechanism can then act. I suggest that a set of behavioral rules including the initiation of a new cell from the groove between two pre-existing cells and the erection of the cell’s walls when the cells base reaches a certain size, are likely involved. The computer simulation shows that a small difference in the set of behavioral rules followed by bees would account for a big difference in the arrangements of cells and consequently on the shape of cells with big consequences in terms of the wax needed to build the comb.

This proposed rule appears to be remarkably similar to that reported by Downing and Jeanne for the wasp *Polistes fuscatus*^18^, despite the different physical properties of the used material and the distant taxonomic relationship, highlighting an interesting case of convergent evolution of the construction behavior pattern.

These reflections may help to bring some clarity in the longstanding debate about the hexagonal shape of the honeycomb cells and provide inputs for more experimental work on the subject as well as stimulating some comparative work on a feature that is shared by other hymenopterans.

## Methods

### Assessing the influence of the pattern of cells on their final shape

Although regularity represents one of the most striking features of the honeycomb, errors can sometimes occur^3^. In order to observe such errors, to see if they can reveal the possible consequences of mismatches involving the reciprocal arrangement of cells, the combs built by an *Apis mellifera ligustica x carnica* colony maintained in the experimental apiary of the Dipartimento di Scienze AgroAlimentari, Ambientali e Animali of the University of Udine, were observed and photographed in the field (Fig. 1a and Fig. S1).

### Estimation of the coefficient of variation of the cell diameter in a honeycomb

To test the hypothesis that the “ruler” of bees (i.e. the body parts used for estimating the distances by bees) is adequate to realize regular cylinders of a given diameter, I measured the coefficient of variation of the cells of a natural comb and compared this figure to that of the bee’s body parts. To do so, five close-up pictures of different portions of a comb were taken and the diameter of cells was measured using ImageJ (Rasband, W.S., ImageJ, U. S. National Institutes of Health, Bethesda, Maryland, USA, http://imagej.nih.gov/ij/, 1997–2015). To minimize possible parallax errors, only the diameter of the six cells surrounding the central one was considered and the coefficient of variation calculated dividing the standard deviation by the average diameter of the six cells. The overall coefficient of variation, reported in the text, is the average of five estimates, whereas the standard deviation describes the variability of such estimates around the reported mean (Supplementary data file 1). Since there is abundant observational evidence about the use of antennae for measuring distances in the honey bee, I measured the length of the flagellum of the antennae of 5 batches of 5 bees, using the “segmented line” tool of ImageJ, as illustrated in Supplementary data file 2, and calculated the coefficient of variation of this morphological feature as described above (Supplementary data file 2).

### Computer simulation of the process of comb construction

According to Downing and Jeanne^18^, the unifying features regarding the regulation of construction behavior are: 1. construction results from a linear series of if-then decisions; 2. cues regulating decision points come from previous construction; 3. the construction behavior pattern is inherited. In this case, the use of computer simulations can help to study the possible ordered patterns emerging from such processes. Therefore, to study the geometry of a comb built according to a certain set of behavioral rules, a spreadsheet was created according to the above-mentioned logic (see Fig. S4 for the flowchart of the program and Supplementary excel file 1). This kind of tool was exploited to make it possible for the interested reader to interpret the behavioral rules that are modeled by looking at the formulas that have been used for designing the spreadsheet. The spreadsheet simulates a two dimensional view of a simplified honeycomb made of quadrangular cells with a fixed dimension (3 × 3 squares); note that, in this way, the progressive enlargement of the virtual comb is constrained in two dimensions. In the spreadsheet, the condition of each square, at each time step is based on the condition of the surrounding squares at the preceding step as modified according to a set of fixed rules, applied in an ordered sequence (Fig. S4). Three conditions are possible for a square: empty or covered with floor (representing the pavement of the bee cells) or wall (representing the walls separating adjacent bee cells). Already filled squares are left untouched (i.e. no rearrangement of square condition is allowed). Construction starts from the top and proceeds laterally and downwards starting with a piece of floor attached to the upper horizontal line, representing the top bar of the virtual comb.

The proposed tool can be regarded as a cellular automaton, similar to those presented by Wolfram^19^; the results obtained here further support the idea that this kind of computer creations can be useful to achieve a better comprehension of some natural processes.

By modifying the settings of the spreadsheet, different rules can be applied with different possible outcomes. To apply the following rules:

(a) New wall is added as soon as the adjacent floor reaches a certain size (in either of both directions),

(b) New floor can be added either to pre-existing floor or walls,

use the following set of criteria for building the floor in Supplementary excel file 1: floor/wall to the left or right: yes; floor above: yes; wall above: yes; vertical stretch of wall above: no. With these settings, a pattern of aligned cells is obtained (Fig. 2a, left column of schemes).

Note that if rule b) is modified such that new floor is added only to pre-existing floor, the construction stops as soon as the first cell is encircled by walls; instead, if lateral growth is permitted either in contact with floor or wall but downward growth is allowed only in contact with pre-existing floor, the construction stops after the first line of cells is built (Supplementary excel file 1 with the following set of criteria for building the floor: floor/wall to the left or right: yes; floor above: yes; wall above: no; vertical stretch of wall above: no).

To note the effect that is obtained downward growth is allowed only between pre-existing cells (in this simplified simulation, this is achieved by allowing the deposition of new floor in contact with the vertical wall separating two adjacent closed cells), use the following set of criteria for building the floor in Supplementary excel file 1: floor/wall to the left or right: yes; floor above: yes; wall above: no; vertical stretch of wall above: yes. In this case, an alternated pattern of cells is obtained (Fig. 2a, right column of schemes).

Of course, the rules regulating the deposition of cell walls can not be changed without completely disrupting the construction process.

To see if a more complete set of rules, but conceptually similar to those presented above, could be exploited to build a more realistic virtual comb made of polygonal cells simulating the circular ones found in nature, another spreadsheet was created. In this case, the rule allowing floor addition in the space between pre-existing cells was implemented with an algorithm such that the distance between the cell walls converging into the groove between existing cells was compared to the cell diameter and new floor was added only in case such distance was smaller (Fig. S7; Supplementary excel file 2).

### Observation of comb construction

To study the construction process of natural combs (Fig. 2b and Fig. S5), a frame with combs under construction was photographed, at two hours intervals, from 10.30 to 16.30, during Summer 2015, after removing the building bees with smoke. The frame was placed in the central portion of a hive containing an *Apis mellifera ligustica x carnica* colony maintained in the experimental apiary of the Dipartimento di Scienze AgroAlimentari, Ambientali e Animali of the University of Udine.

## Additional Information

**How to cite this article**: Nazzi, F. The hexagonal shape of the honeycomb cells depends on the construction behavior of bees. *Sci. Rep.* **6**, 28341; doi: 10.1038/srep28341 (2016).

## Supplementary Material

Supplementay Figures

Supplementary Data File 1

Supplementary Data File 2

Supplementary Excel File 1

Supplementary Excel File 2
