# Supplementary material for: The hexagonal shape of the honeycomb cells depends on the construction behavior of bees
Source: Sci Rep. 2016 Jun 20;6:28341. doi: 10.1038/srep28341 (PMC4913256; doi:10.1038/srep28341)
Supplement: Supplementay Figures [file srep28341-s1.pdf]

SUPPLEMENTARY FIGURES

The hexagonal shape of the honeycomb cells depends on the construction behavior of bees

Francesco Nazzi

Dipartimento di Scienze AgroAlimentari, Ambientali e Animali, Università degli Studi di Udine,

via delle Scienze 206, 33100 Udine, Italy; francesco.nazzi@uniud.it

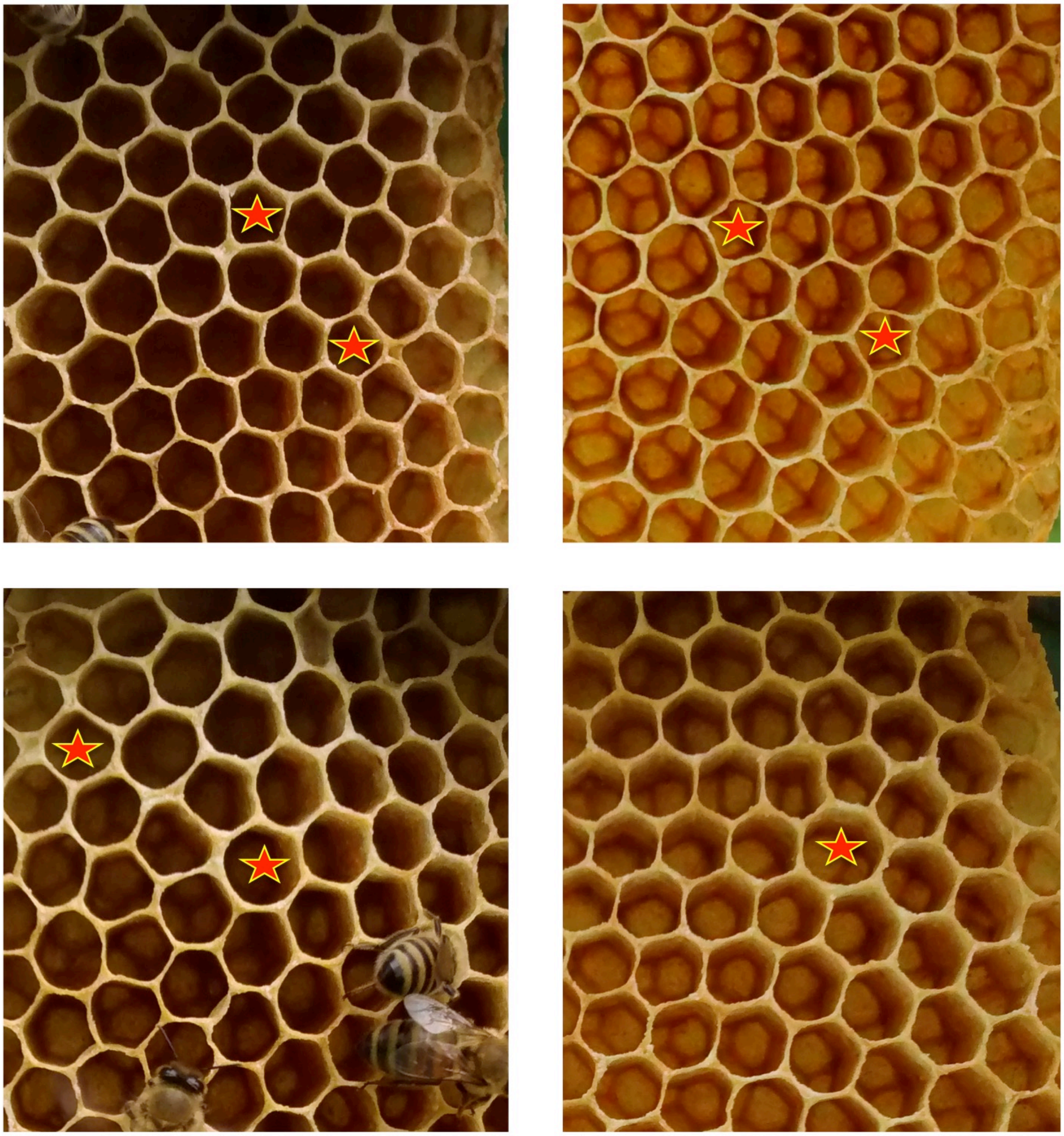

Fig. S1. Building errors in honeycombs and possible consequences. Colored stars mark some cells surrounded by less or more than six other cells; in this case the shape clearly deviates from hexagonality.

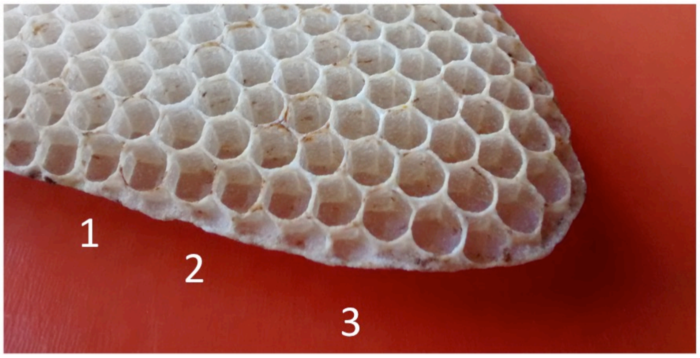

Fig. S2. The margin of a comb with stubs of cells at different stages of construction (1: the construction of the cell base is started in the groove between two pre-existing cells; 2: when the cell base is as large as the cell diameter, the walls are started; 3: the walls encircle the first stub of the cell).

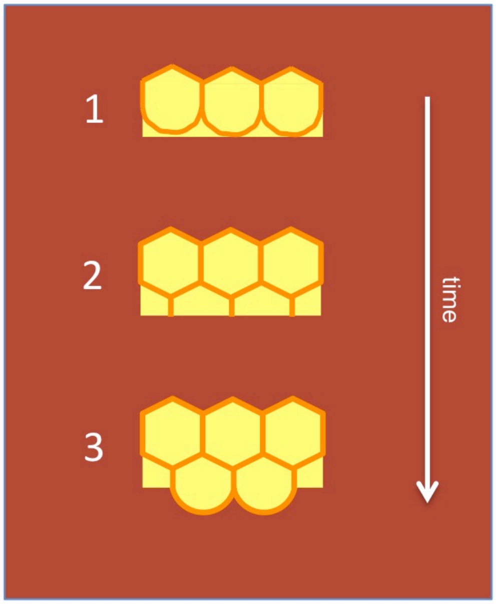

Fig. S3. A schematic representation of the proposed construction process (1: the construction of the cell base is started in the groove between two pre-existing cells; 2: when the cell base is as large as the cell diameter, the walls are started; at this stage the triple junction likely acquires the final form; 3: the walls encircle the first stub of the cell).

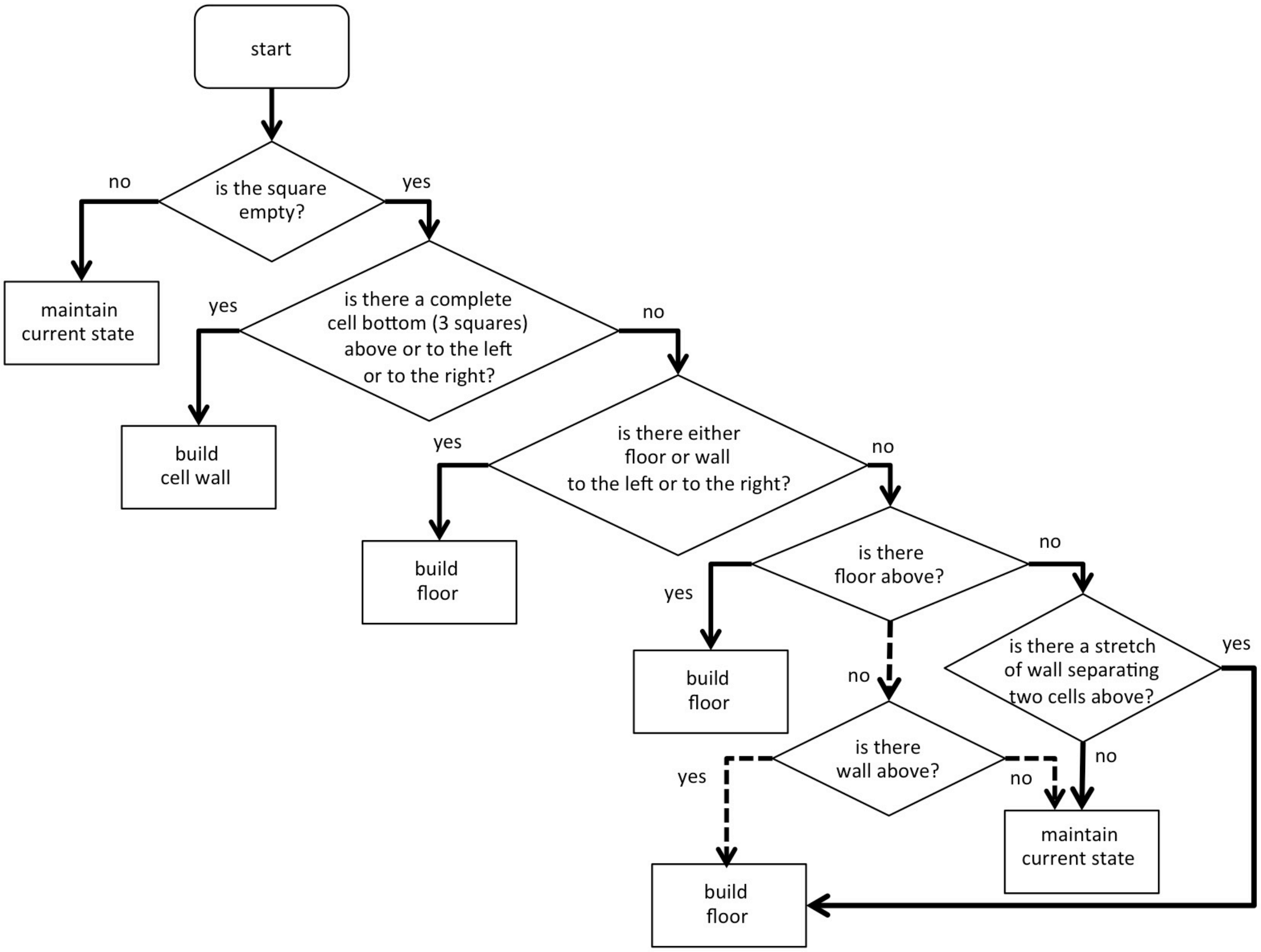

Fig. S4. Flow-chart describing the structure of the spreadsheet used for the simulation of the process of cell construction (Supplementary excel file 1). Solid arrows allow the construction of an alternated pattern of cells; dashed arrows are used to describe an alternative program leading to a pattern of aligned cells.

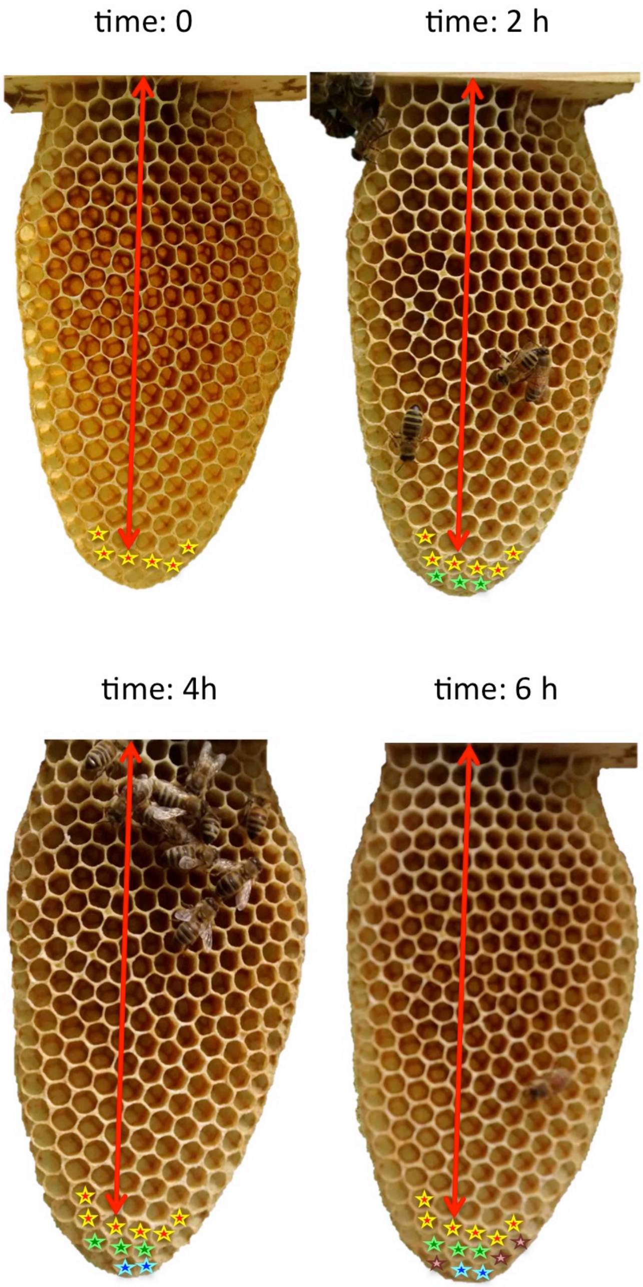

Fig. S5. Progressive growth of the honeycomb. The figure shows four images of a comb taken at 2 hours intervals, showing with different colors the cells added at each step. Red arrows show the scale and facilitate the recognition of the cells at different steps.

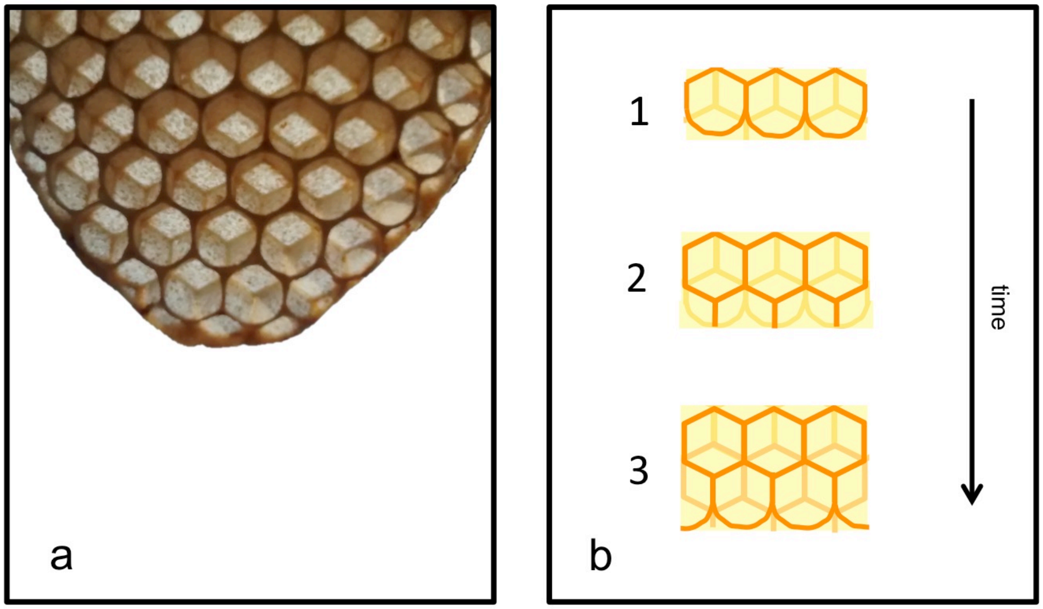

Fig. S6. Matching of the cells on the two sides of the honeycomb. (a) A backlit image of a portion of a honeycomb revealing the typical arrangement of cells on the opposite sides of the comb. (b) A schematic representation of the proposed construction process; the cells of the two sides of the comb are shown with two different color tones.

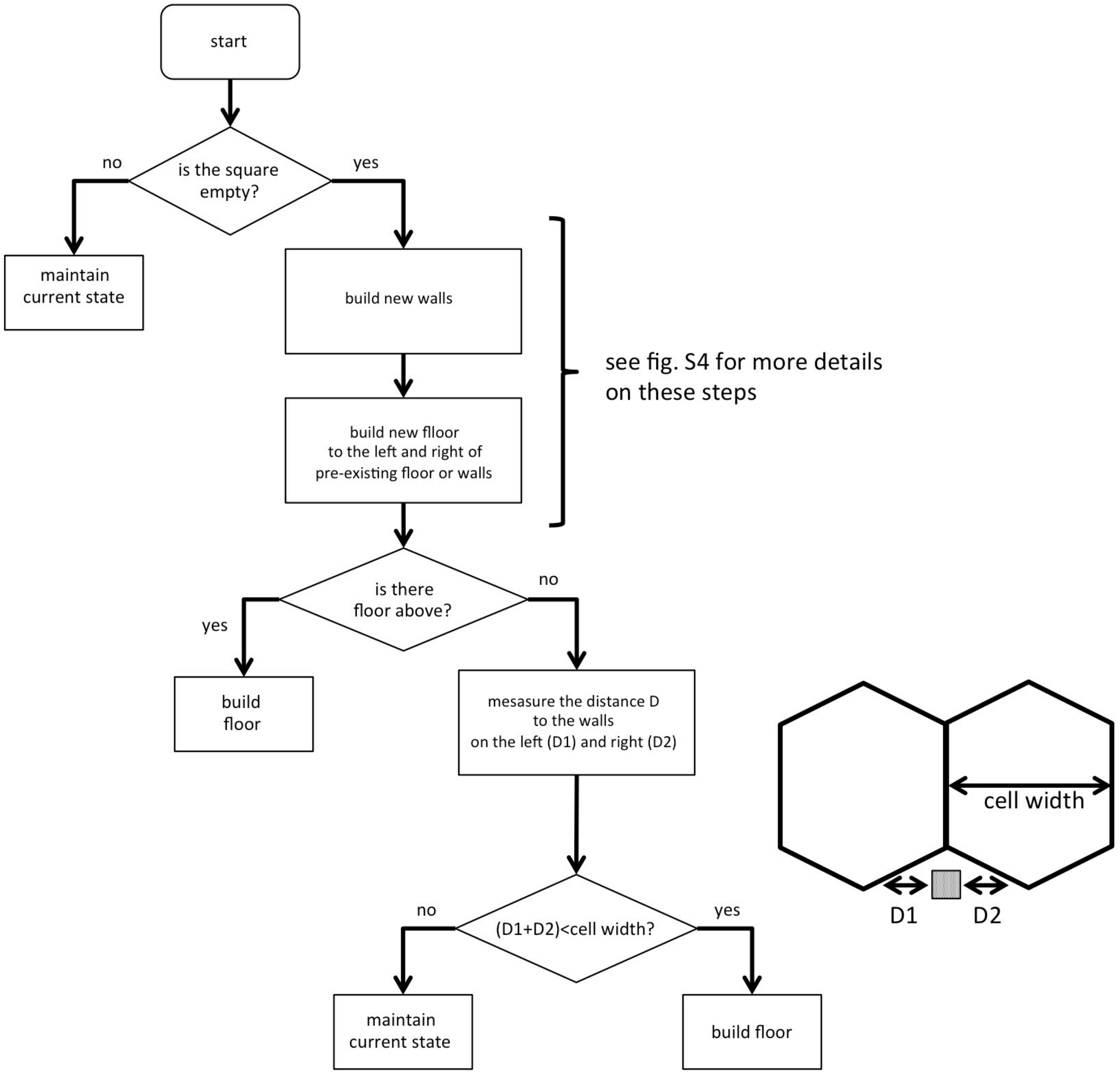

Fig. S7. Simplified flow-chart describing the structure of the spreadsheet used for the simulation of the process of cell construction in Supplementary excel file 2.
